# Successful treatment of allergic bronchopulmonary aspergillosis using a combination of oral voriconazole and the bronchoscopic instillation of amphotericin B: A case report and literature review

**DOI:** 10.1097/MD.0000000000050224

**Published:** 2026-08-28

**Authors:** HuaMan Wu, MaoLiang Tian, Zhao Chen, XiaoYu He, Yang Zeng, Lun Wang, ZhiPing Deng

**Affiliations:** a Department of Respiratory and Critical Care Medicine, Zigong First People’s Hospital, Zigong, Sichuan, China.

**Keywords:** allergic bronchopulmonary aspergillosis, amphotericin B, bronchoscopic instillation, carcinoembryonic antigen, diagnostic criteria, voriconazole

## Abstract

**Rationale:**

Allergic bronchopulmonary aspergillosis (ABPA) is a complex pulmonary disease that results from hypersensitivity reactions triggered in response to *A fumigatus*. To date, there have been few reports of ABPA patients exhibiting elevated serum carcinoembryonic antigen levels. Moreover, while systemic antifungal treatment often results in substantial side effects when administered to ABPA patients, there has been little published regarding bronchoscopic amphotericin B instillation as an alternative treatment strategy.

**Patient concerns:**

We describe an ABPA patient who was repeatedly misdiagnosed with lung cancer. This 60-year-old woman complained of a 5-year history of a recurrent cough with whitish viscous sputum that had recurred and worsened within the 2 days before presentation. This woman had visited hospitals many times during this interval, and pulmonary computed tomography scans had revealed an occupying lesion in the left upper lobe of her lung. This, coupled with her elevated serum carcinoembryonic antigen levels and other lung cancer markers, led to the misdiagnosis of lung cancer.

**Diagnoses:**

As we found that she also exhibited elevated serum IgE and circulating eosinophil counts, and *Aspergillus* was detected through rapid on-site evaluation, we sent a further blood sample for fungal antigen-specific antibody testing. This approach revealed markedly elevated levels of *A fumigatus*-specific IgE, *Aspergillus*-specific IgG and IgM, leading to the confirmation of an ABPA diagnosis.

**Interventions:**

The patient was administered inhaled budesonide together with antifungal therapy consisting of oral voriconazole and bronchoscopic amphotericin B instillation. She was additionally provided empiric antibiotic treatment to alleviate her cough and reduce phlegm production.

**Outcomes:**

Her symptoms and the associated lung lesion improved significantly.

**Lessons:**

Bronchoscopic amphotericin B instillation represents a safe and effective approach to treating ABPA, providing a means of increasing local drug concentrations while minimizing the potential for systemic adverse reactions.

## 1. Introduction

Aspergillosis is an infection caused by the fungus *A fumigatus* and primarily affects the lungs. There are various forms of aspergillosis. Allergic bronchopulmonary aspergillosis (ABPA) is a rare inflammatory disorder that develops due to a hypersensitivity reaction to *A fumigatus*,^[1]^ often in patients with asthma or cystic fibrosis. In *Aspergillus* infections, the cytokines IL-1β, IL-6, and TNF play a crucial and fundamental role.^[2]^ Owing to the atypical clinical symptoms and early imaging changes characteristic of this condition, roughly 50% of ABPA patients are initially believed to have other diseases,^[3]^ including bronchiectasis, bacterial pneumonia, tuberculosis, or lung cancer.^[4]^ Carcinoembryonic antigen (CEA) is a relatively specific biomarker of lung cancer and gastrointestinal tumors, but there have been a few reports of elevated serum CEA concentrations in ABPA patients, increasing their odds of misdiagnosis.

Current approaches to treating ABPA primarily consist of glucocorticoid and antifungal therapies.^[5]^ Relative to oral or intravenous glucocorticoids, inhaled glucocorticoids can achieve effective concentrations within the airways while causing fewer side effects such that they are an ideal first-line approach to treating ABPA.^[6]^ Voriconazole is the preferred drug when treating aspergillosis caused by *A fumigatus*,^[7,8]^ with combination amphotericin B treatment being recommended as appropriate.^[7]^ However, amphotericin B is associated with multisystem adverse reactions.^[9,10]^ To date, there have been very few reports exploring the treatment of ABPA through the bronchoscopic instillation of amphotericin B.

In this report, we describe the case of a patient who was ultimately diagnosed with ABPA following repeated misdiagnosis as lung cancer, and who went into remission following a combination of oral voriconazole and bronchoscopic amphotericin B instillation.

## 2. Case report

**Figure 1.**
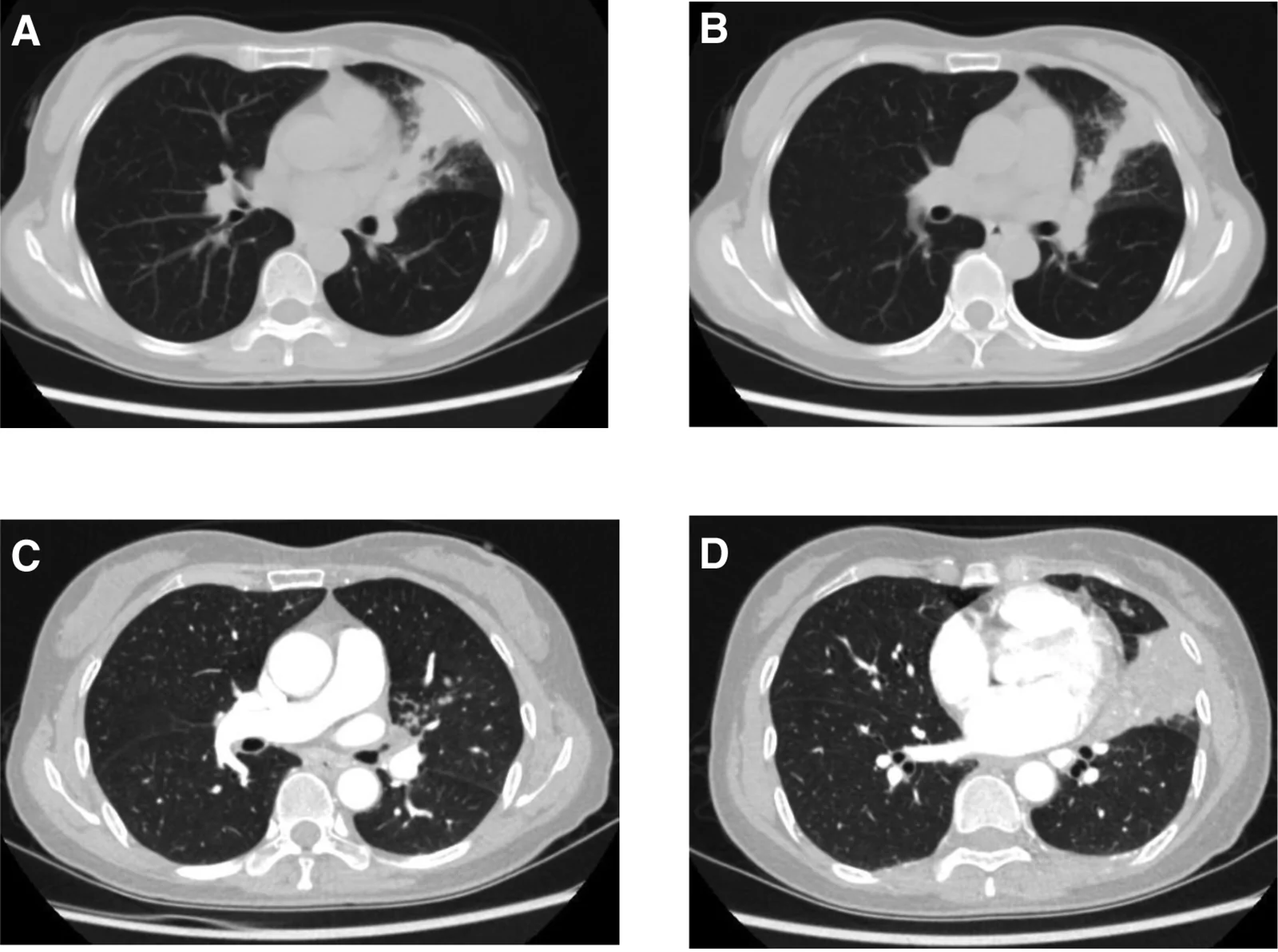
(A) and (B) Chest CT five years ago. (C) and (D) The Chest CT at admission.

A 60-year-old female farmer patient who was a nonsmoker was admitted to our hospital in July 2023 with a 5-year history of a recurring cough and whitish, viscous sputum that had recurred and become aggravated over the last 2 days. Five years previously, she had exhibited an excessive paroxysmal cough and expectoration of sticky sputum without any definite cause. Her presentation was more obvious at night while lying flat, and did not vary seasonally. She did not exhibit any evidence of fever, chills, headache, dizziness, muscular soreness, night sweats, hemoptysis, or chest pain. While the patient reported relief of her symptoms following unspecified symptomatic treatment in a local hospital, they invariably recurred with time such that she was ultimately transferred to our hospital. Pulmonary computed tomography (CT) revealed a soft tissue density shadow at the origin of the left upper lobe bronchus that was accompanied by distal obstructive changes and suspected neoplastic lesions (Fig. 1A–B). She visited additional hospitals, where she was considered to have chronic inflammation or lung cancer.

Two days before her most recent presentation to our hospital, her symptoms had recurred and worsened. Upon admission, no significant change in body weight was observed relative to disease onset. She had a history of chronic non-atrophic gastritis, and her family history and dust exposure were unremarkable. The patient’s body mass index was 16.23kg/m^2^. Physical examination revealed scattered moist rales in both lungs, but no other obvious positive signs. Initial lab tests did not reveal any evidence of an inflammatory syndrome, as her white cell, high-sensitivity C-reactive protein, and erythrocyte sedimentation rate values were within normal ranges. However, her eosinophil count was slightly elevated at 0.82 × 10^9^/L. Arterial blood gas analyses revealed normal pH and PaCO_2_ levels, with a PaO_2_ of 80 mm Hg. Her serum CEA levels were elevated at 7.995 ng/mL, and her levels of carbohydrate antigen (CA)125, CA19–9, and gastrin-releasing peptide precursor were 36.20 U/mL, 85.93 U/mL, and 87.21 pg/mL, respectively. Given the clinical suspicion of lung cancer and obstructive pneumonia, she was administered intravenous cefoxitin and oral acetylcysteine.

**Figure 2.**
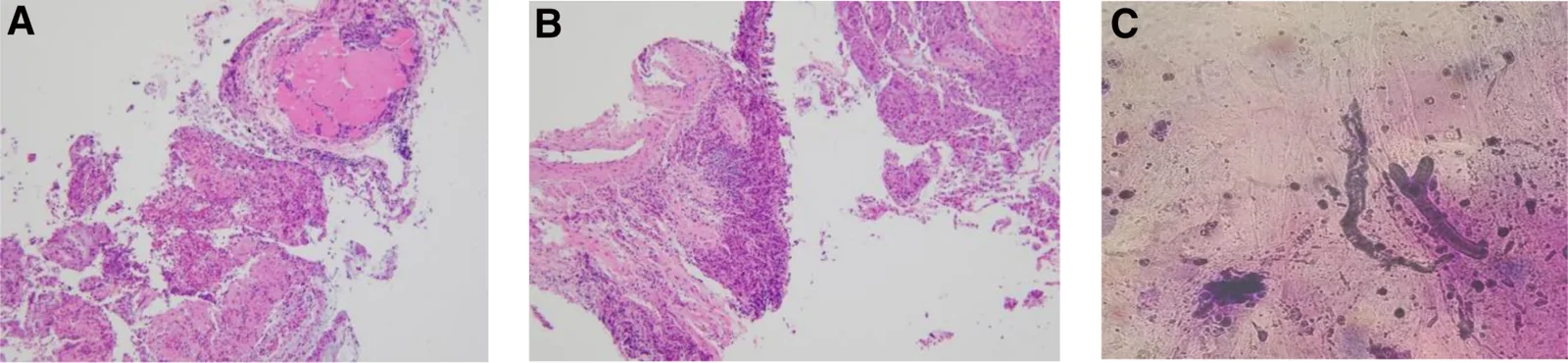
(A) and (B) The pathological examination. (C) The ROSE.

Lung function tests revealed small airway dysfunction. Chest enhanced CT imaging revealed a bronchial cutoff sign in the lingular bronchus of the left upper lobe, with patchy shadows of uneven density with unclear boundaries being visible in the distal side. These changes were significantly advanced when compared to the results of prior CT analyses, underscoring a need to exclude the presence of lung tumors (Fig. 1C–D). Fiberbronchoscopy revealed abundant yellow-white mucus in the left main bronchus and lingular bronchus, with the opening of the lingular bronchus being completely obstructed by mucus. After repeatedly rinsing to remove these secretions, we observed the narrowing of the opening of the lingular bronchus with abundant purulent secretions at the distal end. Bronchoalveolar lavage fluid was negative for M.tuberculosis DNA, non-tuberculosis mycobacterial DNA, and X-pert testing. Bronchoalveolar lavage fluid samples were also sent for metagenomic next-generation sequencing (mNGS) to exclude infection, and the results revealed the absence of any pathogenic microorganisms. Samples were also collected for pathological examination and rapid on-site evaluation (ROSE). While pathological examination only revealed the presence of chronic inflammatory changes (Fig. 2A–B), ROSE led to the identification of septate mycelium branching at 45° angles within these samples (Fig. 2C). As the patient was infected with *Aspergillus*, serum total immunoglobulin (Ig)E concentrations were measured and found to be elevated at 144.55 U/mL (0–100U/mL). Serum samples were also submitted to an external laboratory and found to be positive for *A fumigatus*-specific IgE, *Aspergillus*-specific IgM and IgG; their values were 1.90KUA/L (0.00–0.35KUA/L), 123.29 AU/mL (0.00–79.99AU/mL) and >500 AU/mL (0.00–79.99AU/mL), respectively.

In light of these results, she was definitively diagnosed with ABPA, bronchiectasis with infection, and obstructive pneumonia. Building on the above treatment regimen, she was additionally treated with inhaled atomized budesonide (1mg bid) and oral voriconazole (200mg q12h). Amphotericin B (5mg) was dissolved in 5 mL of sterile water and used for bronchoscopic instillation following the administration of 10 mL of 2% lidocaine aerosol inhalation anesthesia and 5 mL of lidocaine local anesthesia. This procedure was repeated 1 week later.

**Figure 3.**
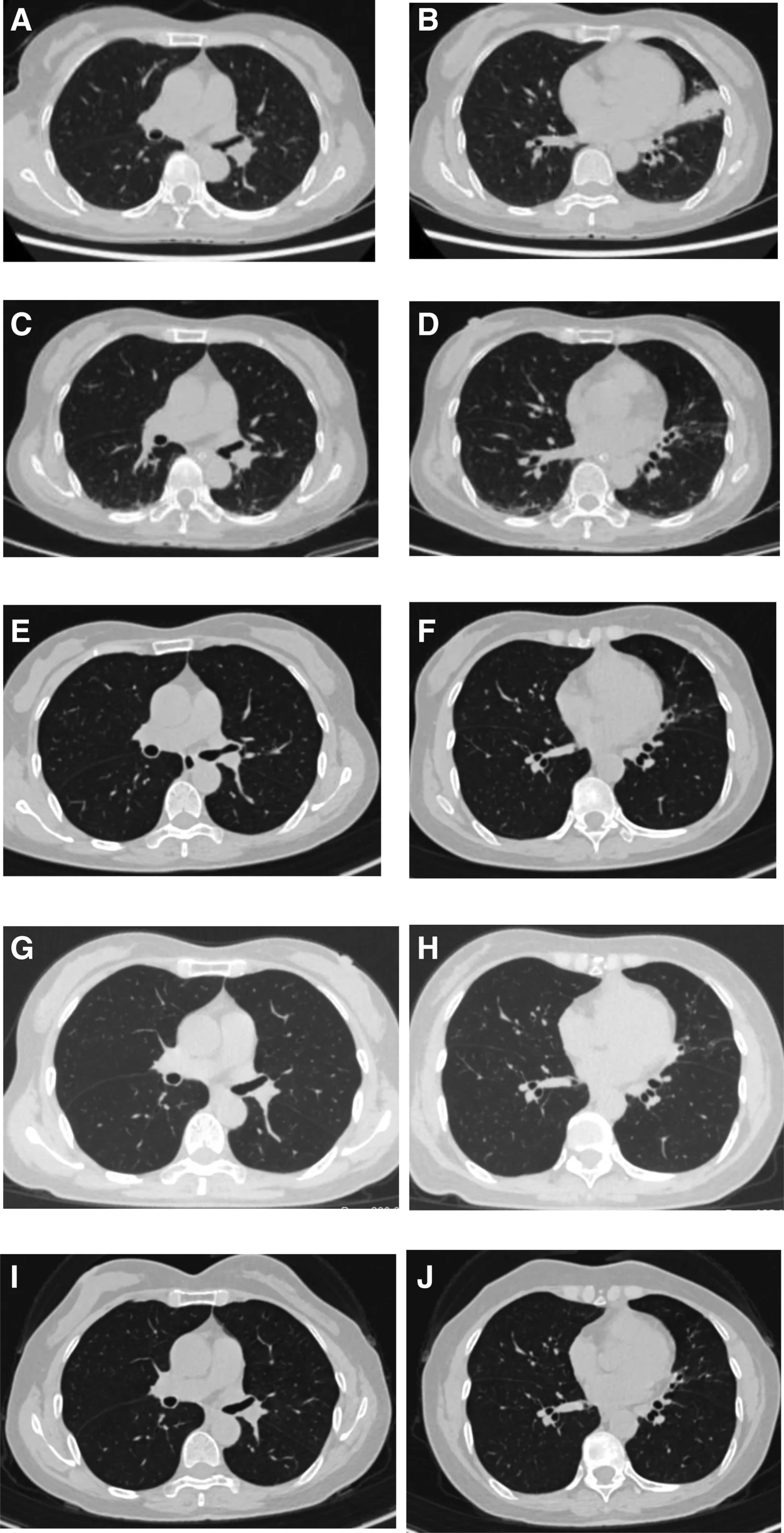
(A) and (B) The Chest CT scans at 2-week treatment. (C) and (D) The Chest CT scans at 1-month post-discharge. (E–H) and (I–J) Chest CT scans at 8, 18, and 36 months after discharge.

Following treatment for 2 weeks, the patient’s symptoms were notably alleviated, and improvements were observed on reexamination. Her PaO_2_ and eosinophil count had returned to normal levels (91 mm Hg and 0.22 × 10^9^/L, respectively), while her CEA, CA125, CA19–9, and gastrin-releasing peptide precursor levels had declined to 4.540 ng/mL, 42.70 U/mL, 37.22 U/mL, and 59.32 pg/mL, respectively. Serum total IgE was 127.19 U/mL (0–100U/mL). Chest CT reexamination similarly revealed significant improvements in the previously noted obstructive changes and bronchial cutoff sign (Fig. 3A–B).

Steroid treatment was modified to methylprednisolone (16 mg qd), and the patient was ultimately discharged with 2 weekly dose reductions of 4 mg. The dose was adjusted for weight; she additionally continued to utilize oral voriconazole 150 mg every 12 hours. Chest CT scans at 1-month post-discharge revealed the disappearance of the bronchial cutoff sign and the near-total absorption of inflammatory lesions (Fig. 3C–D). The patient underwent follow-up chest CT examinations at 8, 18 and 36 months after discharge, which confirmed stable disease conditions (Fig. 3E–J). Post-discharge CEA dropped to 2.933 at month 8 and 1.872 at month 18; IgE declined to 76.55 and 50.12 at the same intervals.

### 2.1. Ethics statement

The studies involving human participants were reviewed and approved by Zigong First People’s Hospital. The patient provided her written informed consent to participate in this study. Written informed consent was obtained from the individual for the publication of any potentially identifiable images or data included in this article.

## 3. Discussion

Correct diagnoses and effective treatments are essential to ensure optimal clinical outcomes. The patient in the present case exhibited a number of distinctive features. Firstly, her clinical presentation was nonspecific, with multiple pulmonary CT scans revealing what were initially suspected to be neoplastic lesions. This, coupled with her elevated levels of CEA and other lung cancer markers, led to her initial misdiagnosis. Secondly, she exhibited an elevated eosinophil count, CT results consistent with central bronchiectasis with obstructive atelectasis, and her mNGS and pathological examination results were all negative for any specific diagnosis. Fortunately, ROSE revealed the potential presence of *Aspergillus,* and we thus submitted further samples for testing of total IgE, *A fumigatus*-specific IgE, *Aspergillus*-specific IgM and IgG, leading to her definitive diagnosis of ABPA. Lastly, this patient benefitted from a treatment regimen consisting of oral voriconazole combined with the bronchoscopic instillation of amphotericin B.

When Aspergillus is inhaled, immune cells resident in the lung, such as alveolar macrophages, recognize the fungus’s Pathogen-Associated Molecular Patterns, activating Toll-Like Receptors. This activation leads to the activation of the mitogen-activated protein kinase pathways.^[11]^ But ABPA does not exhibit any distinctive clinical characteristics, with patients often presenting with chronic cough, sputum production, chest tightness, wheezing, and related symptoms. This disease is often characterized by difficult-to-treat asthma, central bronchiectasis, and recurrent pulmonary infection,^[6,12]^ although some patients are free of any asthma symptoms. Central bronchiectasis may be a characteristic feature of ABPA that can be relatively inconspicuous, as 33%–43% of bronchiectasis extends to the periphery,^[13]^ and its sensitivity when guiding the diagnosis of ABPA is just 37%.^[14]^ These factors can contribute to the delayed diagnosis and treatment of ABPA patients, contributing to further complications such as bronchiectasis with chronic sputum production, atelectasis, pulmonary fibrosis, or loss of lung function.

ABPA has been reported to occur in patients with asthma, cystic fibrosis, chronic obstructive pulmonary disease (COPD), and bronchiectasis.^[15,16]^ In a report published 10 years ago, ABPA was estimated to impact 2.5%–5% of asthma patients,^[17,18]^ but a more recent systematic review estimated a higher prevalence of 11.3%; studies published from India were associated with increased prevalence, possibly due to genetic and other environmental factors.^[19]^ A decade ago, the global burden of ABPA was estimated at 4.8 million, assuming the prevalence of ABPA complicating asthma to be 2.5%,^[17]^ which suggested that the global burden of ABPA is now large. ABPA rates among patients with COPD have also risen from an estimated 1%–2%^[20,21]^ to 9.5% in recent years.^[22]^ The prevalence of ABPA in cystic fibrosis patients varied markedly from 2%–25%,^[23,24]^ with an overall prevalence of approximately 10.1% among adults.^[24]^ Similarly, ABPA impacts roughly 4%–8% of bronchiectasis patients,^[25,26]^ with no apparent trends in these rates over recent decades. ABPA can also rarely arise in immunodeficient patients, including individuals with chronic granulomatous disease, hyper-IgE syndrome, and human immunodeficiency virus infections.^[1]^ The patient in this case was free of these diseases, with her bronchiectasis instead being a complication of her prolonged untreated cases of ABPA. In addition, except for Korea and Japan, where ABPA mainly occurs in the elderly,^[27,28]^ ABPA in other countries mainly occurs in the young and middle-aged adults,^[29,30]^ which also may be influenced by genes, environment and other concomitant diseases.

Elevated CEA levels should generally prompt initial evaluations for lung cancer or tumors of the gastrointestinal tract, followed by further assessment for benign lesions such as lung abscesses or pulmonary interstitial fibrosis. Reports of ABPA patients exhibiting elevated CEA levels are relatively rare, and these individuals face a particularly high risk of misdiagnosis or missed diagnosis. Our patients hadn’t other cause of CEA increased, such as smoking, diabetes, or other gastrointestinal diseases. The specific mechanisms that result in elevated CEA concentrations in ABPA patients have yet to be established, although a report attribute this change to lung inflammation.^[31]^ Chronic ABPA-related inflammation may thus stimulate the production and release of CEA into systemic circulation following alveolar destruction. Alternatively, eosinophils may serve as CEA-secreting cells in these patients.^[32]^ However, no rigorous testing of these hypotheses has yet been performed. CEA levels have been reported to be associated with symptom severity, eosinophil counts, IgE levels, and CT findings in some ABPA patients.^[33]^ However, another report found that CEA levels were only consistent with CT findings, whereas they were unrelated to eosinophil counts or IgE levels.^[31]^ In the present case, CEA levels paralleled changes in PaO_2_, eosinophil counts, presentation, and chest CT findings, whereas they were not consistent with IgE concentrations. When this patient’s CEA levels had declined to nearly normal levels following a 2-week treatment period, her total IgE concentrations remained largely unchanged, suggesting that CEA may be a more sensitive biomarker than IgE when assessing therapeutic efficacy in ABPA patients. However, as these findings are based on a single case, additional research will be essential to gauge the accuracy of these findings.

The diagnostic criteria for ABPA are consistently being updated. The initial diagnostic criteria were proposed by Rosenberg and Patterson in 1977,^[34]^ with more recent updates from Greenberger and Patterson (1988),^[35]^ Stevens (2003),^[36]^ Knutsen (2012),^[37]^ and the International Society for Human and Animal Mycology diagnostic criteria (2013).^[38]^ None of these criteria, however, apply to patients without cystic fibrosis or asthma even though they account for approximately 7%–24% of all ABPA patients.^[3,39,40]^ For this reason, Japanese diagnostic criteria were updated in 2021, yielding significantly higher sensitivity levels when diagnosing ABPA as compared to the criteria established by Rosenberg and the International Society for Human and Animal Mycology (94.4%, 49.2%, and 82.7%, respectively),^[41]^ although its sensitivity for the diagnosis of ABPA patients without asthma declined to 85.7%.^[41]^ China thus established updated expert consensus guidelines for the diagnosis and treatment of ABPA in 2022.^[42]^ These guidelines defined asthma, bronchiectasis, COPD, or cystic fibrosis as predisposing conditions and included 2 mandatory criteria: elevated *A fumigatus*-specific IgE levels or immediate cutaneous hypersensitivity to *A fumigatus*, and elevated total IgE levels. These guidelines also included 3 minor criteria, at least 2 of which should be met to support a diagnosis of ABPA: blood eosinophils count > 0.5 × 10^9^/L, radiographic lung findings consistent with ABPA, and elevated *Aspergillus*-specific-IgG levels. This patient had predisposing factors for bronchiectasis and met 2 major diagnostic criteria (elevated *A fumigatus*-specific IgE and total IgE) and 2 minor diagnostic criteria (1 and 3). In general, pathological examination is unnecessary for the diagnosis of ABPA, our patient’s result didn’t show any specific pathological findings. ROSE is an on-site touch imprint cytology approach that is used to assess the adequacy of biopsy samples and to make a preliminary diagnosis during procedures including transbronchial biopsy, transbronchial needle aspiration, and percutaneous lung biopsy.^[43–46]^ ROSE has been reported to exhibit high rates of sensitivity, specificity, and consistency when used to diagnose lung cancer and specific subtypes thereof.^[47,48]^ It can also be used to effectively diagnose fungal infections,^[49,50]^ yielding results that are highly consistent with histopathological findings. Here, ROSE exhibited clear advantages including its ability to enable timely diagnosis. While mNGS also offers a high level of diagnostic value for lung infections,^[51,52]^ it failed to detect *Aspergillus* in the present case.

Corticosteroid treatment can effectively suppress the excessive inflammation and related damage caused by *Aspergillus* in ABPA patients, but its long duration of being taken orally can contribute to great adverse effects and provide little greater benefit in the rate of recurrence of exacerbations.^[53]^ Inhaled corticosteroid treatment is an excellent oral corticosteroid-sparing approach.^[54]^ As such, aerosolized steroid treatment was administered to our patient. The combination of glucocorticoids and the antifungal triazole can reportedly achieve efficacy superior to that of glucocorticoids alone.^[55]^ Antifungal drugs can alleviate fungal colonization of the airways and related inflammation, thereby relieving symptoms, reducing the need for steroid use, and minimizing the risk of disease recurrence.^[15,55]^ Relative to itraconazole, voriconazole exhibits better gastrointestinal tolerability and bioavailability,^[56]^ and it is thus the preferred first-line antifungal agent.^[57]^ Amphotericin B is the most commonly used antifungal drug, and it is capable of preventing ABPA progression.^[58]^ Intravenous ABPA treatment, however, is associated with substantial renal and cardiac toxicity, and localized tissue necrosis and vascular obstruction in the lungs also limit its efficacy when administered intravenously or through aerosol inhalation. In contrast, the bronchoscopic instillation of amphotericin B can overcome these limitations, yet there have been few studies focused on this treatment approach. However, bronchoscopic amphotericin B instillation has been shown to be effective and safe when used to treat pulmonary fungi.^[59]^ Amphotericin B functions by generating microporous channels on the cell membrane that increase membrane permeability for both fungal and human cells, resulting in the leakage of key bioactive substances and oxidative damage to the cell, culminating in cell death.^[60]^ Local bronchoscopic infusion can achieve higher drug concentrations within target lesions while avoiding the dangers associated with systemic treatment. As such, the combination of voriconazole and amphotericin B was selected to treat the patient in the present case, we hope that provides new perspectives for future treatment.

## 4. Conclusions

In summary, we herein described the case of a woman with ABPA who was repeatedly misdiagnosed with lung cancer owing to her atypical clinical symptoms, elevated CEA levels, and abnormal CT changes. The final diagnosis of ABPA in this case was made based on eosinophil counts, total serum IgE levels, ROSE results, *A fumigatus*-specific IgE, and *Aspergillus*-specific IgG and IgM concentrations. Bronchoscopic local instillation of amphotericin B elevates local drug concentrations in lesions and reduces the risk of systemic adverse reactions. No recurrence was observed during long-term follow-up, indicating that this regimen is safe and feasible for the treatment of ABPA. We hope our experience can provide some insights for clinicians.

## Acknowledgments

We thank all the staff members in Department of Respiratory and Critical Care Medicine for their contributions to patient care.

## Author contributions

**Conceptualization:** HuaMan Wu.

**Data curation:** HuaMan Wu.

**Formal analysis:** HuaMan Wu, XiaoYu He.

**Software:** HuaMan Wu, Yang Zeng.

**Methodology:** MaoLiang Tian, Zhao Chen, Lun Wang, ZhiPing Deng.

**Project administration:** MaoLiang Tian, ZhiPing Deng.

**Investigation:** Zhao Chen, ZhiPing Deng.

**Supervision:** ZhiPing Deng.

**Writing – original draft:** HuaMan Wu.

**Writing – review & editing:** HuaMan Wu.
